# Rifampin- or Capreomycin-Induced Remodeling of the *Mycobacterium smegmatis* Mycolic Acid Layer Is Mitigated in Synergistic Combinations with Cationic Antimicrobial Peptides

**DOI:** 10.1128/mSphere.00218-18

**Published:** 2018-07-18

**Authors:** DeDe Kwun-Wai Man, Tokuwa Kanno, Giorgia Manzo, Brian D. Robertson, Jenny K. W. Lam, A. James Mason

**Affiliations:** a Institute of Pharmaceutical Sciences, School of Cancer & Pharmaceutical Sciences, King’s College London, London, United Kingdom; b Department of Pharmacology & Pharmacy, Li Ka Shing Faculty of Medicine, The University of Hong Kong, Pokfulam, Hong Kong, China; c MRC Centre for Molecular Bacteriology and Infection, Department of Medicine, Imperial College London, London, United Kingdom; University of Nebraska Medical Center

**Keywords:** mycobacteria, NMR metabolomics, capreomycin, rifampin, synergy, tyloxapol

## Abstract

The mycobacterial cell wall affords natural resistance to antibiotics. Antimicrobial peptides (AMPs) modify the surface properties of mycobacteria and can act synergistically with antibiotics from differing classes. Here, we investigate the response of Mycobacterium smegmatis to the presence of rifampin or capreomycin, either alone or in combination with two synthetic, cationic, α-helical AMPs that are distinguished by the presence (D-LAK120-HP13) or absence (D-LAK120-A) of a kink-inducing proline. Using a combination of high-resolution magic angle spinning nuclear magnetic resonance (HR-MAS NMR) metabolomics, diphenylhexatriene (DPH) fluorescence anisotropy measurements, and laurdan emission spectroscopy, we show that M. smegmatis responds to challenge with rifampin or capreomycin by substantially altering its metabolism and, in particular, by remodeling the cell envelope. Overall, the changes are consistent with a reduction of trehalose dimycolate and an increase of trehalose monomycolate and are associated with increased rigidity of the mycolic acid layer observed following challenge by capreomycin but not rifampin. Challenge with D-LAK120-A or D-LAK120-HP13 induced no or modest changes, respectively, in mycomembrane metabolites and did not induce a significant increase in the rigidity of the mycolic acid layer. Furthermore, the response to rifampin or capreomycin was significantly reduced when these were combined with D-LAK120-HP13 and D-LAK120-A, respectively, suggesting a possible mechanism for the synergy of these combinations. The remodeling of the mycomembrane in M. smegmatis is therefore identified as an important countermeasure deployed against rifampin or capreomycin, but this can be mitigated and the efficacy of rifampin or capreomycin potentiated by combining the drug with AMPs.

**IMPORTANCE** We have used a combined NMR metabolomics/biophysical approach to better understand differences in the mechanisms of two closely related antimicrobial peptides, as well as the response of the model organism Mycobacterium smegmatis to challenge with first- or second-line antibiotics used against mycobacterial pathogens. We show that, in addition to membrane damage, the triggering of oxidative stress may be an important part of the mechanism of action of one AMP. The metabolic shift that accompanied rifampin and, particularly, capreomycin challenge was associated with modest and more dramatic changes, respectively, in the mycomembrane, providing a rationale for how the response to one antibiotic may affect bacterial penetration and, hence, the action of another. This study presents the first insights into how antimicrobial peptides may operate synergistically with existing antibiotics whose efficacy is waning or sensitize MDR mycobacteria and/or latent mycobacterial infections to them, prolonging the useful life of these drugs.

## Abstract (precis)

We have used a combined NMR metabolomics/biophysical approach to better understand differences in the mechanisms of two closely related antimicrobial peptides, as well as the response of the model organism Mycobacterium smegmatis to challenge with first- or second-line antibiotics used against mycobacterial pathogens. We show that, in addition to membrane damage, the triggering of oxidative stress may be an important part of the mechanism of action of one AMP. The metabolic shift that accompanied rifampin and, particularly, capreomycin challenge was associated with modest and more dramatic changes, respectively, in the mycomembrane, providing a rationale for how the response to one antibiotic may affect bacterial penetration and, hence, the action of another. This study presents the first insights into how antimicrobial peptides may operate synergistically with existing antibiotics whose efficacy is waning or sensitize MDR mycobacteria and/or latent mycobacterial infections to them, prolonging the useful life of these drugs.

## INTRODUCTION

*Mycobacterium* spp. are responsible for a variety of diseases, including tuberculosis (TB), leprosy, pulmonary disease, lymphadenitis, and skin and disseminated diseases. Although the incidence of tuberculosis is falling globally at a rate of about 2% per year, it still carries the greatest worldwide disease burden, with 10.4 million people falling ill with TB in 2016 and 1.7 million deaths (1). Infections due to nontuberculous mycobacteria are also increasingly recognized (2). Although the reduced global incidence of tuberculosis is welcome, first-line therapies for tuberculosis are increasingly failing, with 600,000 new cases of tuberculosis resistant to the most effective first-line antibiotic, rifampin. Of these, 490,000 cases were multidrug resistant (MDR), and a substantial proportion of these were extensively drug-resistant (XDR). While 95% of tuberculosis deaths occur in low- and middle-income countries (LMICs), tuberculosis is also an increasing problem in the developed world, where reactivation of latent tuberculosis infections is of particular concern (3). Mycobacteria are intrinsically resistant to many antibiotics that are effective against other bacteria, and this is thought to be due to the superior protection offered by both mycobacterial outer (7- to 8-nm thick) and inner (6- to 7-nm) membranes, a layer of arabinogalactan-peptidoglycan (6 to 7 nm), and a periplasm (14 to 17 nm) containing lipomannan and lipoarabinomannan, resulting in a cell envelope between 33 and 39 nm thick (4, 5). The emergence of resistance to the limited number of existing therapies for tuberculosis has focused attention on the particular problems associated with finding drugs capable of breaching the mycobacterial cell wall, since this may offer a means of either directly killing mycobacterial pathogens or of resensitizing them to existing first-line antibiotics.

In this regard, antimicrobial peptides (AMPs) are of considerable interest, since their mechanisms of action are often associated with direct damage to bacterial plasma membranes and/or penetration within the bacterial cytoplasm to access intracellular targets (6), and these actions often result in rapid bacterial cell death—a property that may be desirable in reducing the incidence of resistance to AMPs. Antimicrobial peptides are therefore increasingly being evaluated as antituberculosis agents (7, 8).

Indeed, not only have antimicrobial peptides been evaluated in isolation, combinations of antimicrobial peptides with first-line antimycobacterial agents have already been shown to be effective (6, 9–11). All d-amino acid isomers of these peptides displayed improved stability and enhanced mycobacterial selectivity (12).

Our own research has focused on a series of highly cationic antimicrobial peptides comprised of d-amino acids, rationally designed to adopt α-helix conformations within biological membranes (13). These AMPs have a detergent-like ability to enable disruption of colonies of Mycobacterium tuberculosis strain H37Ra (13), are able to inhibit the growth of MDR and XDR strains of M. tuberculosis when cultured in THP-1 macrophage cells, and potentiate the activity of the first-line antibiotic isoniazid *in vitro* (14). The D-LAK peptides were designed to be cationic and amphipathic, with the angle subtended by the positively charged lysine residues when the peptide adopts an idealized α-helix conformation that is modified to enhance disruption of anionic model membranes (13). Further, the role of conformational flexibility was investigated and proline residues were introduced to disrupt the α-helix. The positioning of this proline kink was shown to be important and could enhance activity against Gram-negative bacteria while also mitigating hemolysis (13). Conformational flexibility is a key property of potent antimicrobial peptides, such as pleurocidin, which is associated with greater penetration of bacterial membranes and the ability to reach intracellular targets (14, 15). A transcriptomic and NMR metabolomic approach to understanding the mechanism of action of D-LAK120-HP13 supports an ability to penetrate within Gram-negative bacteria (16). Interestingly, however, the proline-containing D-LAK peptides did not always outperform their proline-free analogues when tested against Mycobacterium tuberculosis H37Ra, highlighting that the mechanism of action against mycobacteria is likely to be distinct from that which is effective against Gram-negative bacteria. Consequently, our understanding of the structural features that promote antimycobacterial activity is poorly developed.

To address this gap in our understanding, we have investigated in more detail how Mycobacterium smegmatis responds to challenge with proline-free (D-LAK120-A) and proline-containing (D-LAK120-HP13) D-LAK peptides. We examined the activity of and bacterial response to the combination of each peptide with rifampin or capreomycin to investigate the mechanism of the observed, albeit modest, synergy. Testing the hypothesis that the membrane activity of the D-LAK peptides is key to both their antimycobacterial activity and their ability to potentiate the activity of rifampin and capreomycin, we incorporated high-resolution magic angle spinning nuclear magnetic resonance (HR-MAS NMR) metabolomics with fluorescence spectroscopy of M. smegmatis membrane-incorporated *trans*-1,6-diphenyl-1,3,5-hexatriene (DPH) and laurdan dyes, which are sensitive to changes in membrane fluidity and order, respectively. These techniques were able to distinguish between the two D-LAK peptides and revealed that, when challenged with subinhibitory concentrations of rifampin or capreomycin, M. smegmatis remodeled its membrane. These observations suggest why and how antimicrobial peptides may act and may be modified to improve their ability to potentiate existing antimycobacterial treatments.

## RESULTS

### Action of D-LAK peptides on M. smegmatis.

**FIG 1:**
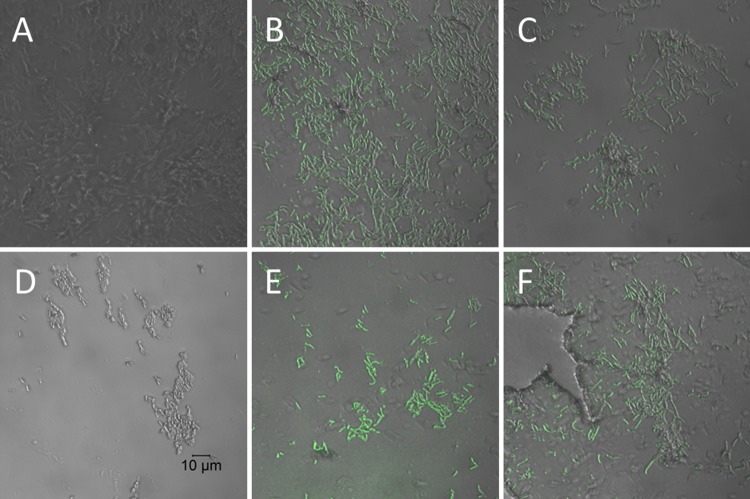
AMPs render Mycobacterium smegmatis permeable to fluorescein isothiocyanate (FITC)-dextran. Confocal microscope images of 1.1 × 10^8^ CFU/ml M. smegmatis mc^2^ 155 either unchallenged (A) or challenged with rifampin alone (D) or D-LAK120-A (B and E) or D-LAK120-HP13 (C and F) at 0.5 MIC (B and C) or 2× MIC (E and F). In contrast to the results for rifampin, both peptides facilitate absorption or adsorption of the 150-kDa fluorescently labeled dextran.

The action of the two D-LAK AMPs on M. smegmatis was first investigated to confirm that, as suspected from their activity against M. smegmatis, the peptides acted on the mycobacterial cell wall. Confocal microscopic investigation of M. smegmatis strain mc^2^ 155 challenged with sub- or supra-MICs of D-LAK120-A, D-LAK120-HP13, or rifampin indicate that both peptides, but not rifampin, cause the bacteria to stain positive with fluorescein isothiocyanate (FITC)-labeled, 150-kDa dextran (Fig. 1). FITC-dextran has been used in a number of studies with vesicles to probe the membrane permeabilization ability of diverse antimicrobial peptides, and a series of labeled dextran conjugates are available to measure the size of pores that may be formed by antimicrobial peptides (17–20). In the case of melittin, dextran of 4 kDa was readily able to escape from loaded vesicles, but much less of the 50-kDa analogue was released. This indicated that a pore with a defined diameter of 25 to 30 Å is formed (17). In contrast, other peptides facilitate the escape of dextrans across the full range of sizes tested, but complete leakage is never achieved (19, 20). These results have been interpreted as evidence for rather large but transient lesions (19). At 150 kDa, the dextran used in the present study is substantially larger than those used in previous studies of escape from lipid vesicles, and it is not possible from images with this resolution to determine whether the labeled dextran has penetrated within the bacterial cells. It is may be that the same functionality that causes D-LAK peptides to prevent aggregation of M. tuberculosis colonies also enables the dextran to adsorb to the surface of M. smegmatis.

**FIG 2:**
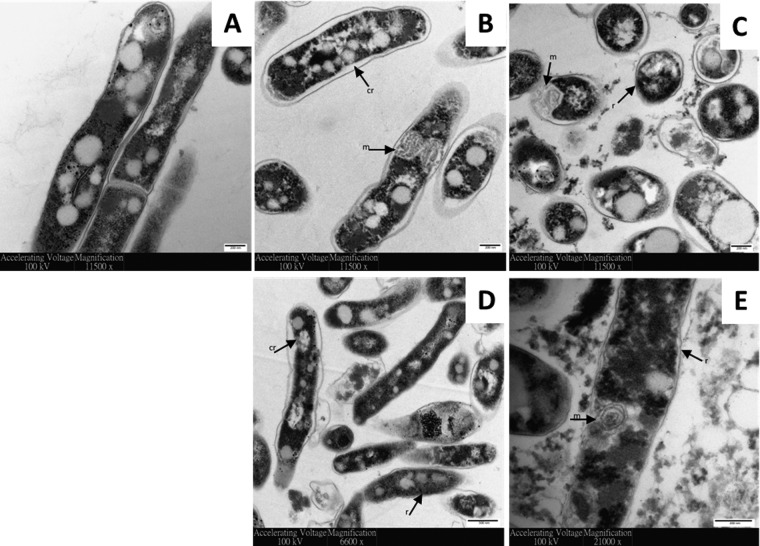
AMPs disrupt membrane surface of M. smegmatis. Transmission electron micrographs of 1.1 × 10^8^ CFU/ml M. smegmatis mc^2^ 155 either unchallenged (A) or challenged with D-LAK120-A (B and C) or D-LAK120-HP13 (D and E) for 5 (B and D) or 30 (C and E) min. r, membrane ruffling; cr, cytoplasmic retraction; m, mesosome.

Transmission electron microscopy (TEM) was exploited to visualize the effect of D-LAK peptides on the ultrastructural changes of M. smegmatis (Fig. 2). Unchallenged cells have characteristic lipid inclusions, a regular rod-shaped cell body with well-defined intact cell membranes, and homogeneous cytoplasm (Fig. 2A). The cell envelope was distinctly visible, with cytosol bound by the plasma membrane, surrounded by an internal and then an outer electron-dense layer (21, 22). The cell content was slightly pulled away in some control cells, which is attributed to the sample preparation process. Membrane ruffling was observed after treating M. smegmatis mc^2^ 155 at 1× MIC with D-LAK120-A (Fig. 2B) or D-LAK-120-HP13 (Fig. 2D) for 5 min. Cytoplasmic retraction was observed as the inner cell membrane detached from the outer cell wall. This phenomenon was reported when Escherichia coli and Bacillus subtilis were exposed to antimicrobial peptides (23). Similar findings have been observed for exposure of Mycobacterium tuberculosis (Erdman strain) to antimicrobial peptides; granulysin demonstrated inner cell membrane detachment as observed in osmotic lysis (24). The reduction in membrane uniformity suggests a cell-penetrating ability for the D-LAK peptides. Peptide treatment also induced notable intracellular changes, with clumped cytosol and an increased amount of mesosome formation. Mesosome structures have been observed previously in bacteria challenged by antimicrobial peptides (25, 26). The formation of mesosomes was suggested as a repair mechanism by bacteria to combat cell lysis (27). Prolonged treatment (30 min) with D-LAK peptides resulted in large-scale cell lysis and the presence of cell debris (Fig. 2C and E). This indicates that the fate of bacteria challenged with inhibitory concentrations of D-LAK peptides is disturbed cell membranes and cell wall disintegration, membrane rupture, and release of intracellular contents.

### Activity of D-LAK peptides against M. smegmatis alone or in combination with rifampin or capreomycin.

All four antibiotics were tested in isolation against M. smegmatis mc^2^ 155 both in the presence and absence of tyloxapol, a nonionic liquid polymer which is often included when growing mycobacteria in planktonic suspension, to prevent clumping (Table 1). As a detergent, it can be expected to disrupt membranes and remove components of the cell envelope and may facilitate the entry of some antimicrobial drugs. As such, we have studied the behavior of M. smegmatis mc^2^ 155 with and without this common additive to the growth medium. Notably, both peptides and rifampin were more potent when tyloxapol was present in the culture medium, but the activity of capreomycin was unchanged.

**TABLE 1:** MICs of D-LAK peptides, rifampin, and capreomycin against M. smegmatis mc^2^ 155 when used alone or in combination, in the presence or absence of tyloxapol

Antimicrobial measured (antimicrobial used in combination)	Mean MIC_50_ ± SE (µM) with:	
	Tyloxapol	No tyloxapol
Rifampin	19.9 ± 3.04	38.1 ± 5.83
Capreomycin	1.72 ± 0.43	1.35 ± 0.25
D-LAK120-A	0.82 ± 0.07	1.68 ± 0.43
D-LAK120-HP13	0.63 ± 0.03	1.80 ± 0.39
Rifampin (D-LAK120-A)	9.33 ± 4.38	59.8 ± 1.39
Rifampin (D-LAK120-HP13)	8.88 ± 1.44	14.6 ± 2.91
Capreomycin (D-LAK120-A)	0.58 ± 0.24	0.46 ± 0.18
Capreomycin (D-LAK120-HP13)	0.52 ± 0.28	0.67 ± 0.28
D-LAK120-A (rifampin)	0.91 ± 0.05	1.56 ± 0.17
D-LAK120-A (capreomycin)	0.09 ± 0.01	0.62 ± 0.07
D-LAK120-HP13 (rifampin)	0.75 ± 0.22	0.77 ± 0.08
D-LAK120-HP13 (capreomycin)	0.14 ± 0.01	0.85 ± 0.15

Combinations of rifampin or capreomycin with each of the D-LAK peptides were then tested (Table 2). In the absence of tyloxapol, the combinations of capreomycin and the D-LAK peptides are modestly synergistic. Effective inhibition is achieved by D-LAK120-A and capreomycin when used in combination, with approximately a third of each agent required to achieve the same effect as when each agent is used alone. The effect is more marked when the combination is used in the presence of tyloxapol, with a fractional inhibitory concentration (FIC) of <0.5, which is widely accepted as a threshold for synergism. Under these conditions, only approximately 1/10 of the amount of D-LAK120-A is required for the same effect. A similar effect is seen when capreomycin is used in combination with D-LAK120-HP13, but the synergistic effect is more modest in both the presence and absence of tyloxapol. Rifampin demonstrates no synergy with D-LAK120-A, and these antibiotics may even be antagonistic when no tyloxapol is added to the growth medium. In contrast, though modestly, D-LAK120-HP13 may assist the activity of rifampin, and a little less than half as much rifampin is required to achieve the same inhibitory effect as when rifampin is used alone. Analogous experiments were performed with isoniazid, but no synergism was detected with either peptide, and hence, these combinations were not studied further in the present work.

**TABLE 2:** FICs of D-LAK peptides combined with rifampin or capreomycin against M. smegmatis mc^2^ 155 in the presence or absence of tyloxapol

Antimicrobial combination	Mean FIC ± SE with:	
	Tyloxapol	No tyloxapol
Rifampin + D-LAK120-A	1.58 ± 0.10	2.50 ± 0.14
Rifampin + D-LAK120-HP13	1.64 ± 0.03	0.81 ± 0.02
Capreomycin + D-LAK120-A	0.45 ± 0.13	0.71 ± 0.05
Capreomycin + D-LAK120-HP13	0.53 ± 0.20	0.97 ± 0.20

### DPH anisotropy and laurdan fluorescence indicate substantial changes in membrane physical properties when M. smegmatis is cultured in the presence of capreomycin or tyloxapol.

DPH fluorescence polarization or fluorescence anisotropy analysis is commonly used to determine changes in bacterial cytoplasmic membrane fluidity under environmental stress (28).

**FIG 3:**
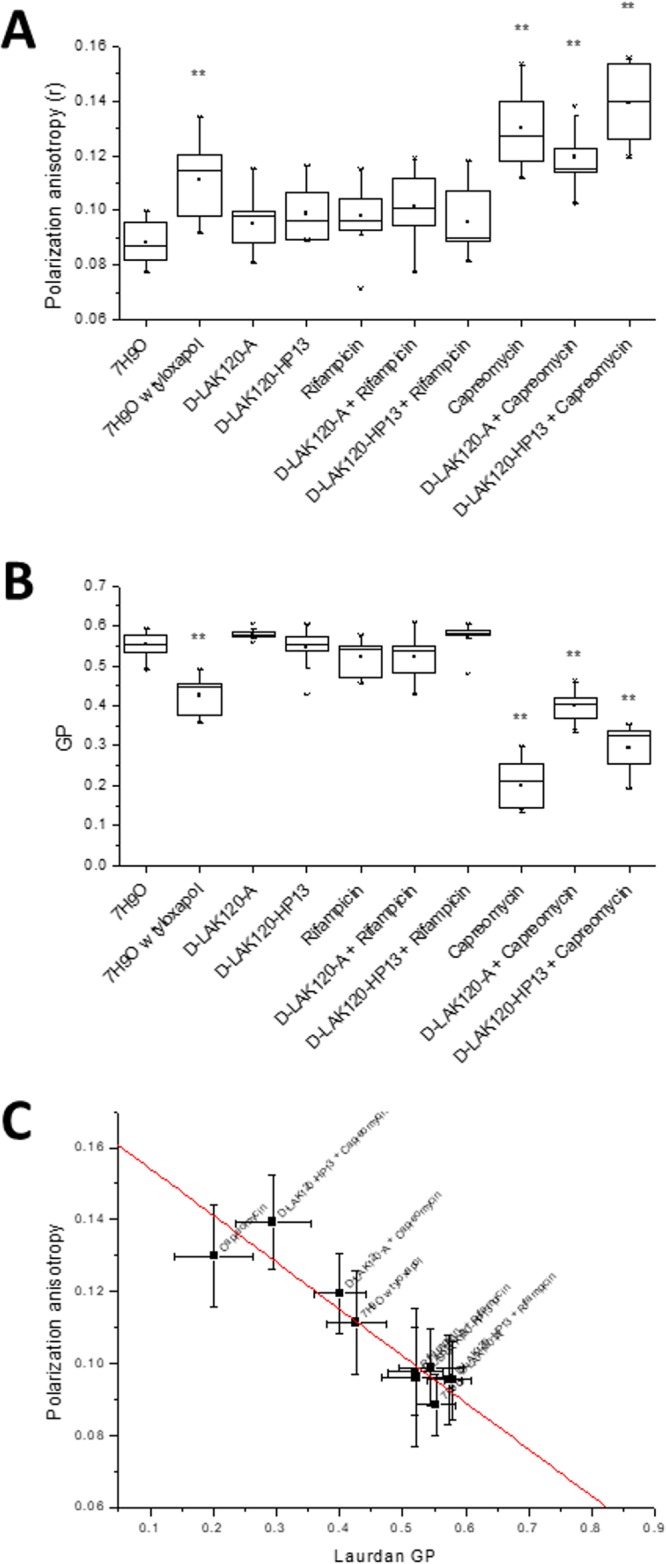
Fluorescence spectroscopic perspective of the response of M. smegmatis mc^2^ 155 to challenge with antibiotics. Data are shown for DPH fluorescence anisotropy (A), laurdan GP fluorescence (B), and correlation (*R*^2^ = 0.856) of the results for DPH and laurdan (C). **, *P* < 0.05 with respect to the results for 7H9O (growth media alone).

As a polyene hydrophobic dye, DPH localizes to the hydrophobic core of lipid bilayers. It aligns parallel to the acyl chains of lipids, and hence, its ability to reorient is dependent on the packing of its lipid neighbors. A high degree of orientation and, hence, fluorescence anisotropy results from a more rigid lipid bilayer. Significant increases (*P* < 0.05) in DPH fluorescence polarization anisotropy (*r*) were observed for M. smegmatis mc^2^ 155 challenged during growth with 0.75 MIC or FIC of capreomycin or its combinations with D-LAK120-A or D-LAK120-HP13 (Fig. 3A) or when grown in the presence of 0.025% tyloxapol, the latter producing a more modest increase. Small increases in fluorescence anisotropy were observed when bacteria were challenged with the D-LAK peptides or with rifampin, either alone or in combination with the D-LAK peptides, but these were not significant compared with the results for unchallenged bacteria. Only challenge with capreomycin alone or in combination with either D-LAK peptide causes a substantial increase in membrane rigidity in M. smegmatis.

Laurdan is a solvatochromic fluorescent dye which inserts into lipid bilayers with the fatty acid chain embedded in the hydrophobic core of the bilayer and the *N*,*N*-dimethyl-2-napththylamine in the more polar interfacial region. The dipole moment between the dimethylamino and carbonyl moieties is sensitive to local changes in polarity, which will shift the emission maximum. Calculating the difference in emission at 440 and 490 nm generates a generalized polarization (GP) value from which membrane order can be inferred. As a lipid bilayer becomes disordered, more water can penetrate the interfacial region, which becomes more polar, and the GP value decreases (29).

The GP value is therefore a measure of disorder in the lipid bilayer interfacial region, although other factors may influence the polarity of the environment in which the fluorophore is located and some caution is advisable when interpreting laurdan GP in biological systems. Nevertheless, laurdan generalized polarization has been used in, e.g., Bacillus subtilis to detect the effect of challenge with a synthetic cyclic hexapeptide, cWFW (30), and analyzing the organization of the bacterial membrane by flotillins (31) and MreB (32). Significant changes (*P* < 0.05) in laurdan GP were observed for M. smegmatis mc^2^ 155 challenged during growth with 0.75 MIC or 0.75 FIC of capreomycin or its combination with D-LAK120-A or D-LAK120-HP13 (Fig. 3B). Again, a change in membrane properties was detected for bacteria incubated with tyloxapol, but a noticeable reduction in GP following challenge with rifampin is nonsignificant. The change in GP for bacteria challenged with combinations of capreomycin is of a lower magnitude than that observed when the bacteria are challenged with capreomycin alone, but the differences between these three conditions are nonsignificant. Notably, the GP values under each of the conditions were reduced relative to those of the unchallenged bacteria, which implies that the local environment of the laurdan probe is becoming more polar, something that would normally be associated with an increase in disorder in the interfacial region. Since the increase in DPH anisotropy for these conditions is associated with an increase in membrane rigidity, a less ordered but more rigid membrane is perhaps surprising. The strong correlation between the DPH and laurdan data (Fig. 3C) does, however, indicate that the two techniques are reporting on the same event. The DPH dye would be expected to reside deep within the hydrophobic core of the membrane, while the laurdan probe would be much closer to the aqueous surface. Consequently, it is possible that the result of M. smegmatis responding to the presence of capreomycin is to modify the hydrophobic core of the membrane, reducing fluidity, while the interfacial region becomes more disordered. While the precise locations of both dyes in the mycobacterial cell membranes are yet to be determined, it can be concluded that the physical properties of the bacterial envelope are substantially altered in response to challenge with capreomycin but not with either rifampin or the D-LAK peptide.

### HR-MAS ^1^H NMR metabolomics reveals substantial changes in M. smegmatis membrane components.

**FIG 4:**
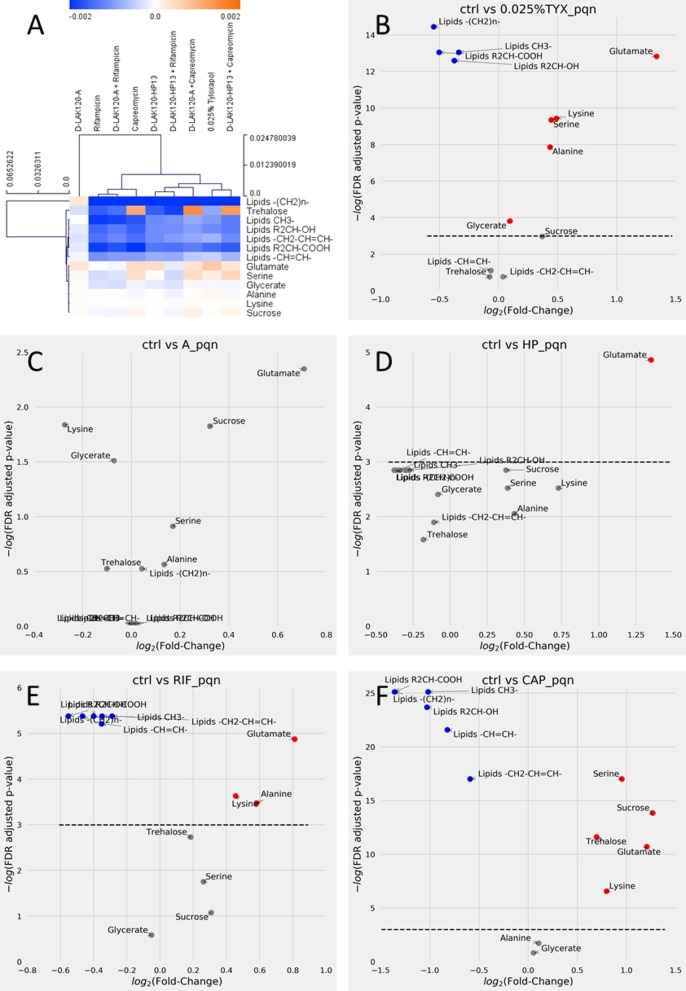
Capreomycin and rifampin induce substantial changes in mycolic acid lipid components in M. smegmatis. (A) Hierarchical clustered heatmap comparing loadings obtained from cross-validated OPLS-DA of ^1^H HR-MAS NMR spectra of M. smegmatis mc^2^ 155 grown under the indicated conditions. (B to F) Volcano plots are shown for individual comparisons of unchallenged bacteria and those challenged with 0.025% tyloxapol (B), D-LAK120-A (C), D-LAK120-HP13 (D), rifampin (E), or capreomycin (F). Volcano plots are of PQN normalized data and allow comparison of fold changes and significance for each metabolite. Blue, significant reductions; red, significant increases; gray, nonsignificant changes in the indicated metabolites.

**FIG 5:**
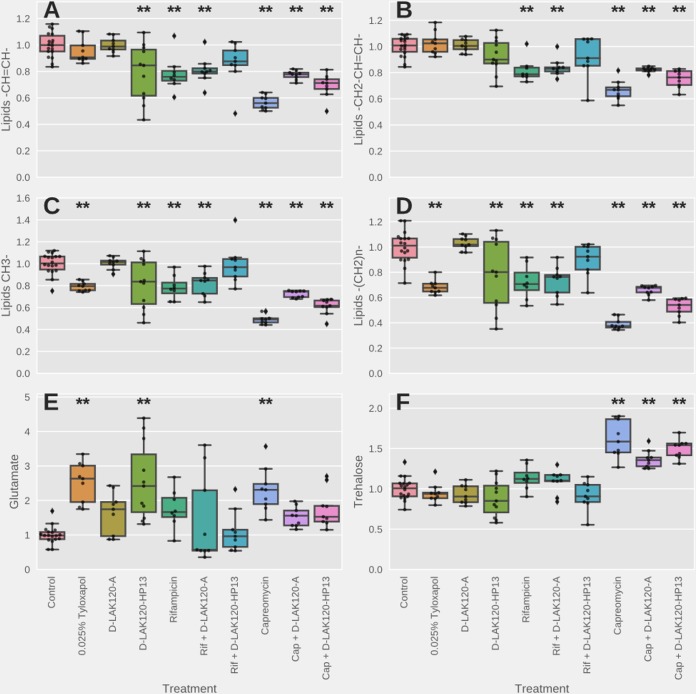
Univariate analysis of relative metabolite levels in M. smegmatis mc^2^ 155 under the indicated conditions. Significant differences with respect to the results for unchallenged bacteria, as determined by one-way ANOVA with Tukey’s *post hoc* test, are indicated (**). Other significant differences are described in the text.

Cross-validated orthogonal projections to latent structures discriminant analysis (OPLS-DA) was used to identify significant changes in ^1^H HR-MAS spectra obtained for M. smegmatis mc^2^ 155 when challenged with 0.75 MIC of individual antimycobacterial drugs, 0.75 FIC of their combinations, or 0.025% tyloxapol. The technique could determine significant differences, as determined by *Q*^2^ (quality assessment statistic) (Table 3), for each model in which loadings were compared in a hierarchical clustered heatmap (Fig. 4A). Volcano plots for individual comparisons allow the identification of the magnitude and significance of changes in individual metabolites (Fig. 4B to F), while changes in key metabolites are compared across treatments to show the impact of each peptide, rifampin, capreomycin, and their combinations (Fig. 5).

**TABLE 3:** Test and permutated *Q*^2^ (quality assessment statistic) scores for OPLS-DA models of ^1^H HR-MAS spectra of M. smegmatis mc^2^ 155^a^

Treatment	*Q*^2^	*Q*^2^ (permutated)
Tyloxapol (0.025%)	0.866	−0.377
D-LAK120-A	0.769	−0.423
D-LAK120-HP13	0.729	−0.360
Rifampin	0.581	−0.426
Rifampin + D-LAK120-A	0.763	−0.410
Rifampin + D-LAK120-HP13	0.430	−0.429
Capreomycin	0.881	−0.347
Capreomycin + D-LAK120-A	0.775	−0.367
Capreomycin + D-LAK120-HP13	0.823	−0.344

a Comparison of test and permutated *Q*^2^ scores for cross-validated OPLS-DA models comparing ^1^H HR-MAS spectra of unchallenged M. smegmatis mc^2^ 155 with those obtained for bacteria under the indicated conditions.

Culturing M. smegmatis mc^2^ 155 in the presence of 0.025% tyloxapol causes significant and substantial changes in several metabolites and components of the mycobacterial membrane (Fig. 4B). An increase in the amount of glutamate in the cells is the greatest change, while there are highly significant reductions in lipid resonances associated with mycolic acid, including the saturated alkyl chains –(CH_2_)_*n*_- and both R_2_CH-COOH and R_2_CH-OH. Notably, resonances associated with unsaturated alkyl groups –CH=CH- and -CH_2_-CH=CH- are unaffected (Fig. 4B and 5A) to D). Mycobacterial mycolic acids comprise a long-branch *mero* chain of 40 to 60 carbons and a short α branch of typically 24 carbons. The shorter α chain is completely saturated. *Mero*-mycolic acids in other mycobacteria contain cyclopropanated mycolic acids, but these are uncommon in M. smegmatis, where the major mycolic acids are a homologous α′ series containing just a *cis*-alkene in the *mero* chain (33–37). An appreciation of the composition of mycolic acids in various mycobacteria can be obtained from two recent reviews (33, 34).

Since the fold changes in all four lipid resonances are similar, this is consistent with a reduction in the number of saturated α chains, with *mero*-mycolic acids, which contain unsaturated hydrocarbons, unaffected. Many of these differences are also observed when M. smegmatis mc^2^ 155 is challenged with either rifampin or capreomycin (Fig. 4E and F).

However, the magnitudes of the responses vary substantially, and there are also qualitative differences, most notably since both rifampin and capreomycin cause reductions in resonances associated with unsaturated alkyl chains. It is notable that the fold changes in the differing lipid resonances are again similar. This is consistent with a reduction in the number of intact mycolic acid molecules rather than a shortening in the length of the mycolic acid alkyl chain. Since resonances for both saturated and unsaturated hydrocarbons are reduced, as well as those for the R_2_CH-OH and R_2_CH-COOH protons, a general reduction in mycolic acid can be inferred.

Mycolic acids are an important component of the mycomembrane. The inner leaflet is formed of mycolic acid linked to arabinogalactan, which is in turn linked to peptidoglycan. The outer leaflet is also formed of lipids based on mycolic acid, but these are considered free to diffuse laterally. These lipids are trehalose dimycolate (TDM) and trehalose monomycolate (TMM). Interestingly, only challenge with capreomycin induces any change in signal intensity attributable to trehalose (Fig. 4F). The significant increase is of a magnitude similar to the decrease observed for the mycolic acid resonances, indicating that the ratio of mycolic acid to trehalose is substantially altered in M. smegmatis following challenge with capreomycin. This would be consistent with a shift in the balance of TDM and TMM, to favor the latter, in the outer leaflet of the mycomembrane.

The scale of the reduction in the mycolic acid resonances caused by capreomycin (Fig. 4F) is much greater than that caused by rifampin (Fig. 4E) or even tyloxapol (Fig. 4B). When changes in mycolic acid lipid resonances are compared across the various challenges (Fig. 5A to D), it is clear that capreomycin has a substantial impact on those from both saturated and unsaturated hydrocarbons and that, while significant (*P* < 0.05), the reductions in intensity due to rifampin are much more modest. Similarly, tyloxapol causes a modest reduction in resonances from saturated hydrocarbons (Fig. 5C and D) but has no significant effect on those from unsaturated hydrocarbons (Fig. 5A and B). Unlike capreomycin, neither rifampin nor tyloxapol induces a significant increase in resonances attributable to trehalose (Fig. 5F). The remodeling of mycolic acid induced by rifampin and tyloxapol is therefore much subtler than that induced by capreomycin, and this is presumably the origin of the nonsignificant changes in membrane physical properties detected as described above using fluorescence techniques.

The response of M. smegmatis mc^2^ 155 to challenge with 0.75 MIC of the two D-LAK peptides is quantitatively and qualitatively different from the response to either rifampin or capreomycin. Although the OPLS-DA models for challenge with either peptide indicate a significant response when all metabolites are considered (Table 3; Fig. S3 in the supplemental material), the magnitudes of changes in individual metabolites are low and few changes pass a stringent significance threshold (Fig. 4C and D). When comparing individual metabolites across the various conditions, both peptides trigger an increase in glutamate in the bacteria (*P* < 0.05); a similar effect is seen for capreomycin but not rifampin (Fig. 4E). In contrast with the results for capreomycin, neither peptide triggered any change in trehalose (Fig. 5F). The proline-containing and proline-free peptides can themselves be distinguished by the absence of any changes in lipid resonances in response to D-LAK120-A, while significant (*P* < 0.05) reductions in lipid resonances are observed for both unsaturated and saturated hydrocarbons in response to D-LAK120-HP13 (Fig. 5A to D).

HR-MAS ^1^H NMR data were also obtained for M. smegmatis mc^2^ 155 challenged with combinations of each D-LAK peptide and either rifampin or capreomycin. For synergistic combinations, the amount of each antibiotic was substantially lower than that used for each antibiotic alone. Modest synergism exists between D-LAK120-A and capreomycin, but there is indifference or even antagonism between D-LAK120-A and rifampin. D-LAK120-A mitigates the reduction in saturated and unsaturated hydrocarbons and increase in trehalose due to capreomycin (*P* < 0.05) (Fig. 5A to D; Fig. S1A and S2A and B), but not the increase in glutamate (Fig. 5E). The responses of M. smegmatis mc^2^ 155 to rifampin and rifampin plus D-LAK120-A are indistinguishable (Fig. 5; Fig. S1C and S2).

10.1128/mSphere.00218-18.1 FIG S1  D-LAK peptides mitigate capreomycin- and rifampin-induced changes in mycolic acid lipid components in M. smegmatis. Volcano plots are shown for individual comparisons of unchallenged bacteria and those challenged with capreomycin plus D-LAK120-A (A), capreomycin plus D-LAK120-HP13 (B), rifampin plus D-LAK120-A (C), or rifampin plus D-LAK120-HP13 (D). Volcano plots are of PQN normalized data and allow comparison of fold changes and significance for each metabolite. Blue, significant reductions; red, significant increases; grey, nonsignificant changes in the indicated metabolites. Download FIG S1, TIF file, 2.7 MB. Copyright © 2018 Man et al. 2018 Man et al. This content is distributed under the terms of the Creative Commons Attribution 4.0 International license.

10.1128/mSphere.00218-18.2 FIG S2  Univariate analysis of relative metabolite levels in M. smegmatis mc^2^ 155 under the indicated conditions. Data are shown for bacteria either unchallenged (0.0), grown in 0.025% tyloxapol (1.0), or challenged during growth with 0.75 MIC D-LAK120-A (2.0), D-LAK120-HP13 (3.0), rifampin (4.0), rifampin plus DLAK120-A (5.0), rifampin plus D-LAK120HP-13 (6.0), capreomycin (7.0), capreomycin plus D-LAK120-A (8.0), or capreomycin plus D-LAK120-HP13 (9.0). Data are shown for lipid –CH2-COOH (A), lipid –CH2–OH (B), serine (C), and alanine (D). Significant differences as determined by one-way ANOVA with Tukey’s *post hoc* test are described in the text. Download FIG S2, TIF file, 2.5 MB. Copyright © 2018 Man et al. 2018 Man et al. This content is distributed under the terms of the Creative Commons Attribution 4.0 International license.

10.1128/mSphere.00218-18.3 FIG S3  OPLS-DA comparisons of HR-MAS ^1^H NMR spectra of M. smegmatis mc^2^ 155 under various challenges. Back-scaled loadings for challenge with 0.025% tyloxapol (A), D-LAK120A (B), or D-LAK120-HP13 (C). Score plots for each comparison are shown in the inset. Download FIG S3, TIF file, 0.5 MB. Copyright © 2018 Man et al. 2018 Man et al. This content is distributed under the terms of the Creative Commons Attribution 4.0 International license.

10.1128/mSphere.00218-18.4 FIG S4  OPLS-DA comparisons of HR-MAS ^1^H NMR spectra of M. smegmatis mc^2^ 155 under various challenges. Back-scaled loadings for challenge with rifampin (A), rifampin and D-LAK120A (B), or rifampin and D-LAK120-HP13 (C). Score plots for each comparison are shown in the inset. Download FIG S4, TIF file, 0.3 MB. Copyright © 2018 Man et al. 2018 Man et al. This content is distributed under the terms of the Creative Commons Attribution 4.0 International license.

10.1128/mSphere.00218-18.5 FIG S5  OPLS-DA comparisons of HR-MAS ^1^H NMR spectra of M. smegmatis mc^2^ 155 under various challenges. Back-scaled loadings for challenge with capreomycin (A), capreomycin and D-LAK120A (B), or capreomycin and D-LAK120HP13 (C). Score plots for each comparison are shown in the inset. Download FIG S5, TIF file, 0.3 MB. Copyright © 2018 Man et al. 2018 Man et al. This content is distributed under the terms of the Creative Commons Attribution 4.0 International license.

In combination susceptibility testing, D-LAK120-HP13 has a mostly additive effect when used in combination with capreomycin but experiences modest synergism with rifampin. D-LAK120-HP13 has a weaker impact on metabolic changes induced by capreomycin or rifampin. Although the magnitudes of reduction of lipid resonances in bacteria treated with D-LAK120-HP13-plus-capreomycin combinations were consistently lower than that observed when capreomycin was used alone (Fig. 5A to D; Fig. S1B and S2A and B), the mitigation was in no case significant. In contrast, although the magnitudes of changes induced by rifampin were smaller, significant mitigation (*P* < 0.05) was observed for lipid -CH_3_ (Fig. 5C), while for other metabolites, the combination of D-LAK120-HP13 and rifampin did not induce significant changes with respect to the results for the unchallenged bacteria (Fig. 5A, B, and D). Notably, the volcano plot of the individual comparisons indicates that no metabolites were significantly changed when this combination was used (Fig. S1D), in contrast to the results for rifampin (Fig. 4F) or, indeed, D-LAK120-HP13 when used alone (Fig. 4D).

### Increased membrane rigidity is associated with altered composition of the mycomembrane.

**FIG 6:**
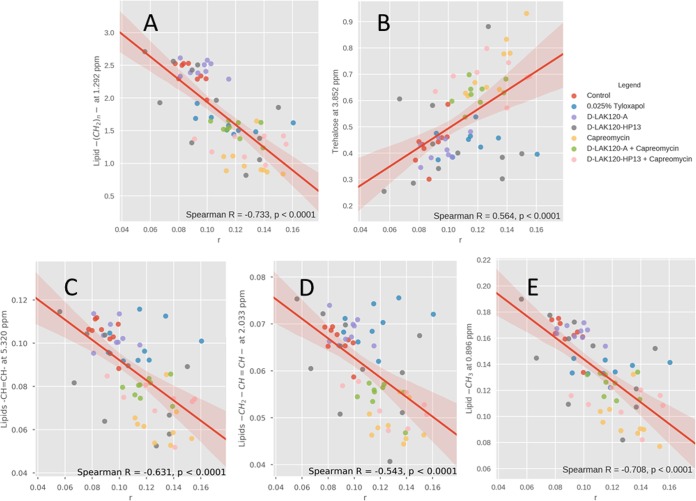
Increased membrane rigidity is associated with altered composition of the mycomembrane. Spearman correlations are shown between DPH anisotropy (*r*) and ^1^H HR-MAS NMR resonance intensity data obtained for M. smegmatis mc^2^ 155 grown without challenge or with 0.025% tyloxapol or 0.75 MIC D-LAK120-A, D-LAK120-HP13, capreomycin, capreomycin plus D-LAK120-A, or capreomycin plus D-LAK120-HP13. Data are shown for lipid –(CH_2_)_*n*_- (A), trehalose (B), lipid –CH=CH- (C), lipid –CH_2_-CH=CH- (D), and lipid -CH_3_ (E).

To better understand the contributions of the various changes in metabolite concentrations to the physical properties of the mycomembrane following challenge with capreomycin and/or D-LAK peptides or growth in the presence of 0.025% tyloxapol, Spearman correlations between individual metabolites and the fluorescence anisotropy data recorded for the same samples were determined using partial least-squares regression (Fig. 6; Fig. S6). Significant (*P* < 0.0001) correlations were detected between fluorescence anisotropy data and each of the metabolites in which substantial changes resulted from challenge with capreomycin, D-LAK120-HP13, or growth in the presence of 0.025% tyloxapol. The strongest of these correlations were negative correlations with saturated hydrocarbons (Fig. 6A and E), while weaker negative or positive correlations were detected with, respectively, unsaturated hydrocarbons (Fig. 6C and D) or trehalose (Fig. 6B). The strength of these correlations may reflect the impact of each component of the mycomembrane in determining its fluidity, or this may reflect the observation that, while all three types of resonance are affected by challenge with capreomycin, D-LAK120-HP13 affects only lipid resonances and tyloxapol affects only saturated hydrocarbons in the shorter α chain. Nevertheless, the increased rigidity of the mycomembrane appears to be a direct result of the observed changes in its composition.

10.1128/mSphere.00218-18.6 FIG S6  PLS regression of ^1^H HR-MAS data obtained for M. smegmatis mc^2^ 155 challenged with capreomycin, D-LAK peptides, or combinations thereof with DPH fluorescence anisotropy measurements obtained for the corresponding samples. (A) Scatterplot showing distance between predicted (red) and true (blue) values for each sample in the regression models. (B and C) Histograms showing the distribution of *R*^2^ (B) and *Q*^2^ (C) model assessment statistics from PLS models. (D) Back-scaled loading plot showing NMR peaks that correlate with DPH fluorescence anisotropy data. Download FIG S6, TIF file, 0.3 MB. Copyright © 2018 Man et al. 2018 Man et al. This content is distributed under the terms of the Creative Commons Attribution 4.0 International license.

### Rifampin but not capreomycin induces detectable changes in metabolite composition of spent medium.

Spent bacterial culture supernatants were also analyzed by OPLDS-DA (Fig. S7). Comparison of the models indicates that only under those conditions where rifampin was present was any qualitative change in M. smegmatis metabolism detected (Fig. S7A). A binary comparison of spent medium from M. smegmatis grown without challenge to fresh medium reveals that the bacteria consume glucose and produce tartrate and 2-hydroxyisobutyrate (Fig. S7B). When challenged with rifampin, there were slight increases in glucose and citrate consumption but substantially reduced production of lactate, alanine, valine, and pyruvate and more modest reductions in the production of malate and tartrate (Fig. S7C). These changes were muted when rifampin was applied in combination with D-LAK120-HP13, where increased consumption of glutamate was also observed (Fig. S7D). Under no other conditions were any changes in individual metabolites detected that passed the significance threshold.

10.1128/mSphere.00218-18.7 FIG S7  Rifampin induces a change in metabolic strategy in M. smegmatis. (A) Hierarchical clustered heatmap comparing loadings obtained from cross-validated OPLS-DA of ^1^H NMR spectra of M. smegmatis mc^2^ 155 spent cultures grown under the indicated conditions. (B to D) Volcano plots are shown for individual comparisons of unchallenged bacteria and either fresh medium (B), bacteria challenged with rifampin (C), or bacteria challenged with rifampin and D-LAK120-HP13 (D). Volcano plots are of PQN-normalized data and allow comparison of fold changes and significance for each metabolite. Blue, significant reductions; red, significant increases; grey, nonsignificant changes in the indicated metabolites. Download FIG S7, TIF file, 2.7 MB. Copyright © 2018 Man et al. 2018 Man et al. This content is distributed under the terms of the Creative Commons Attribution 4.0 International license.

## DISCUSSION

The combination of HR-MAS ^1^H NMR metabolomics and fluorescent probes sensitive to changes in membrane fluidity and order reveals modest changes in the mycomembrane of M. smegmatis mc^2^ 155 in response to challenge with rifampin but much more dramatic changes in response to challenge with capreomycin. The D-LAK peptides cannot be distinguished based on any change in the physical properties of the mycomembrane, but changes in its composition reflect distinct mechanisms for the proline-free and proline-containing analogues. The combinations of both D-LAK peptides with capreomycin are synergistic in the presence of tyloxapol, but when M. smegmatis is cultured in the absence of tyloxapol, this is attenuated, leaving only modest synergy and only with D-LAK120-A and not D-LAK120-HP13; only the latter is modestly beneficial when combined with rifampin. These findings may provide a rationale for understanding the properties required for D-LAK peptides and related molecules to potentiate the activities of first- and second-line treatments against mycobacterial infections and understand whether there is more to the activity of either peptide than enhancing penetration of the mycomembrane.

### Capreomycin acts by inhibiting protein synthesis but induces substantial remodeling of the mycomembrane.

The antibiotic activity of capreomycin against both M. smegmatis and M. tuberculosis is ascribed to its ability to inhibit protein translation by interfering with the function of ribosomes (38). Specifically, capreomycin binds across the ribosomal subunit interface using *tlyA*-encoded methylations in both 16S and 23S rRNAs (39). A transcriptomic approach to understanding the mechanism of action of capreomycin in M. tuberculosis confirmed the importance of this mechanism but additionally revealed substantial changes in a variety of other gene classes, including genes involved in lipid metabolism, cell wall and cell processes, and intermediary metabolism and respiration (40). Notably, capreomycin was shown to affect the glyoxylate shunt, an alternate pathway to the TCA cycle, with upregulation of *icl* (Rv0467, encoding isocitrate lyase), *glcB* (Rv1837c), and *aceAa* (Rv1915), presumably stimulating a process where fatty acids become an important carbon source. There was reduced expression of a block of genes associated with the electron transport chain, coding for NADH dehydrogenase or NADH-ubiquinone oxidoreductase, as well as *gdh*, a probable NAD-dependent glutamate dehydrogenase. Though performed in M. smegmatis, the present study is consistent with these findings; glutamate is seen to accumulate following challenge with capreomycin (and also both D-LAK peptides), while the substantial reduction in mycolic acid is consistent with fatty acids being diverted for use as a carbon source; notably, in contrast with the results for rifampin challenge, no significant changes in individual metabolite levels in spent culture medium were detected. There are some important differences in both the composition of the mycomembrane (33, 34) and the metabolism of these two and other *Mycobacterium* species. Nevertheless, taken together, the two studies indicate that there is likely a substantial shift in metabolism following capreomycin challenge in both M. smegmatis and M. tuberculosis, resulting in greater consumption of fatty acids, depletion of mycolic acid, and rigidification of the mycomembrane. As such, this response from mycobacteria to capreomycin challenge affords a means of altering the barrier that other antimicrobials may have to cross to reach their targets.

### Rifampin acts by inhibiting transcription and induces modest remodeling of the mycomembrane.

The antibiotic activity of rifampin is attributed to its ability to inhibit bacterial-DNA-dependent RNA polymerase activity. M. tuberculosis is more susceptible to rifampin than M. smegmatis, with the latter enjoying a higher level of baseline resistance due to rifampin ADP ribosyltransferase, an enzyme capable of inactivating rifampin (41). However, exposure of rifampin-sensitive M. tuberculosis H37Rv to rifampin induces changes in the expression of genes in the same functional classes affected by capreomycin challenge, i.e., lipid metabolism, cell wall and cell processes, and intermediary metabolism and respiration (42). Furthermore, rifampin, as well as isoniazid and streptomycin, triggers the activation of isocitrate lyases in M. tuberculosis (43), suggesting that the glyoxylate shunt is a general means of overcoming oxidative stress (44). Antimicrobial peptides, including LL-37 and pleurocidin, have been shown to induce oxidative stress in Escherichia coli and Candida albicans, respectively, (45, 46). The modest changes in mycolic acids induced by challenge with rifampin or D-LAK120-HP13 may therefore be indicative of oxidative stress and reflect penetration of the bacterium; notably, D-LAK120-A does not have this effect. The response of M. smegmatis to rifampin differs from that to capreomycin not only quantitatively in the HR-MAS study but qualitatively in the study of spent culture composition, where evidence for an alternate strategy for overcoming oxidative stress emerges. This would be consistent with M. smegmatis altering its metabolism in response to rifampin in a way similar to that used in response to capreomycin but less reliant on the metabolism of fatty acids.

### Distinct membrane activities underpin synergistic combinations.

For antibiotics to have additive or synergistic effects when used in combination, they need to avoid both interactions that reduce the amount of each agent reaching their target(s) and the triggering of responses in the bacteria that counter the activity of the other agent. For combinations of antibiotics to have synergistic effects, they additionally need to act on different targets or have distinct effects on the same target, such that the effect of each individual antibiotic is enhanced. The HR-MAS ^1^H NMR approach enables the investigation of whether antibiotics do indeed differ in their effects on the mycomembrane. This is achieved by obtaining a bacterial perspective of the challenge and inferring differences in the activity of each antibiotic from differences in the response of the bacteria to each challenge. Capreomycin, rifampin, the D-LAK peptides, and tyloxapol each induce qualitatively and/or quantitatively different responses from M. smegmatis, but there is nevertheless considerable overlap.

Tyloxapol improves the potency of rifampin and D-LAK peptides when used alone, but the combination of D-LAK peptides and rifampin is indifferent in the presence of tyloxapol. Since both rifampin and tyloxapol induce membrane remodeling in M. smegmatis, one might speculate that rifampin and tyloxapol combine to produce substantial change in the mycomembrane that inhibits the ability of D-LAK peptides to disrupt or penetrate the membrane. In the absence of tyloxapol-dependent improvements in rifampin potency, the change in membrane properties induced by rifampin alone may differentially affect the two AMPs. This may therefore explain why combinations of rifampin with D-LAK120-A fare worse, indeed being antagonistic, than those containing D-LAK120-HP13. One hypothesis could be that the proline modification of the latter enables this analogue to better penetrate the mycomembrane.

Both D-LAK peptides interact synergistically with capreomycin, albeit modestly. The interaction is enhanced, however, when M. smegmatis is cultured in the presence of 0.025% tyloxapol. While tyloxapol enhances the action of both peptides and rifampin, this is not the case for capreomycin (within the margin of error). It is unlikely, therefore, that tyloxapol improves access for capreomycin, though it may perform this function for either D-LAK peptide. Although the difference between the two peptides in terms of their interaction with capreomycin is small, D-LAK120-A consistently outperforms D-LAK120-HP13. Both peptides seemingly have a similar effect on capreomycin activity, but the poorer synergy between D-LAK120-HP13 and capreomycin may be related to the ability of both agents to trigger changes in the mycomembrane. Since both capreomycin and rifampin trigger changes in the mycomembrane composition, albeit with very different magnitudes of response, this argument seemingly runs counter to the idea that membrane interactions will determine the ability of such molecules to interact synergistically against M. smegmatis. The answer to this may lie in the distinct physical properties of capreomycin and rifampin. Capreomycin is a cationic molecule with a physiological net positive charge of four. Rifampin is also cationic, but its physiological charge is only one. Both molecules must penetrate the bacteria to exert their main antibiotic effects, and hence, the D-LAK peptides, with a net positive charge of nine and a higher affinity for anionic components of the mycomembrane, may provide an important screen for interactions between the membrane and either capreomycin or rifampin. Capreomycin, with the higher charge, would benefit more from this screen, and this may underlie the observed synergy, rather than any induced change in the biophysical properties of the mycomembrane.

### Conclusion.

The combination of HR-MAS ^1^H NMR and fluorescence spectroscopy reveals how the remodeling of the mycomembrane in response to antibiotics, most notably capreomycin, may affect their ability to penetrate or act in combination with other antibiotics against mycobacteria. Furthermore, the approach can distinguish the activities of two D-LAK peptide analogues, based on the different responses of M. smegmatis to AMP challenge and combinations of AMPs and rifampin or capreomycin. Understanding how mycobacteria respond to antibiotics, in particular by remodeling the mycomembrane through which many antibiotics must pass to be effective, may be essential in designing effective antibiotic combinations.

## MATERIALS AND METHODS

Mycobacterium smegmatis mc^2^ 155 was grown in Middlebrook 7H9 broth (catalog number 271310; Difco, Detroit, MI) enriched with 10% (vol/vol) oleic acid-albumin-dextrose-catalase (OADC) (catalog number 212240; Becton, Dickinson, Franklin Lakes, NJ) and 0.5% (vol/vol) glycerol (catalog number G-5516; Sigma-Aldrich) in 50-ml Falcon tubes at 37°C without shaking. A concentration of 0.025% (vol/vol) tyloxapol surfactant (catalog number T8761; Sigma-Aldrich) was added as one of the treatments. Rifampin (RIF; catalog number R3501) and capreomycin sulfate (CAP) from Streptomyces capreolus (catalog number C4142) were from Sigma-Aldrich. D-AMPs D-LAK120-A (KKLALALAKKWLALAKKLALALAKK-NH_2_) and D-LAK120-HP13 (KKALAHALKKWLPALKKLAHALAKK-NH_2_) were supplied by China Peptides Co., Ltd. (Shanghai, China). The purity of synthesized peptides was above 80%, and the peptides were used as supplied. Both peptides were amidated at the C terminus.

### MIC and FIC.

The MICs of the anti-TB agents (rifampin, D-AMPs, and capreomycin) against M. smegmatis mc^2^ 155 were determined using the broth microdilution assay in 96-well plates. Twofold serial dilutions in duplicate were made in Middlebrook 7H9 broth supplemented with OADC and glycerol. An inoculum at an optical density at 600 nm (OD_600_) of 0.02 was prepared by diluting mid-log cultures, and 100 µl was added in each well (~1 × 10^5^ CFU). Amounts of 100 µl each of mycobacterial suspension and anti-TB agent were added in each well to obtain the inoculum concentration of 5 × 10^4^ CFU/ml. Growth controls using no drug and sterile medium were prepared in each assay. The plates were then incubated at 37°C for 24 h before adding 30 µl of 0.02% (wt/vol) resazurin dye, and the color change was evaluated after incubation for another 24 h.

The fractional inhibitory concentrations (FICs) of the combination treatments of rifampin or capreomycin and D-AMPs were determined using a checkerboard assay (47). Synergistic interactions between anti-TB agents in combination treatments were denoted by the fractional inhibitory concentration (FIC) index. The FIC values of the combinations were calculated from the following formula: FIC index = FIC_*A*_ + FIC_*B*_ = [*A*]_in combination_/MIC_*A*_ + [*B*]_in combination_/MIC_*B*_.

### Confocal microscopy.

M. smegmatis mc^2^ 155 (1.1 × 10^8^ CFU/ml) at mid-log phase (OD = 0.6) was treated with 250 µg/ml FITC-labeled, 150-kDa dextran (catalog number 46946; Sigma-Aldrich) and a D-AMP (D-LAK120-A or D-LAK120-HP13) or rifampin at the respective MIC for 20 min. Following treatment, the bacterial cells were centrifuged at 7,500 rpm for 10 min and washed three times with phosphate-buffered saline (PBS). Cells were then fixed in 2% formaldehyde at room temperature (RT) for 20 min. After fixation, the cells were washed twice and resuspended in PBS. Amounts of 20 µl of sample were then allowed to air dry on microscope slides overnight. Samples were then imaged with a 65× oil immersion objective lens using a Zeiss LSM-510 inverted confocal microscope (Carl Zeiss, Inc.). FITC was excited with a 488-nm laser and detected with a 505-nm long-pass filter.

### TEM.

M. smegmatis mc^2^ 155 (1 × 10^7^ CFU/ml) at mid-log phase (OD = 0.6) was treated with D-LAK120-A and D-LAK120-HP13 at their respective MICs for 5 and 30 min. Samples were washed three times with PBS, and cell pellets were collected by centrifugation. Cell pellets were then fixed in 2.5% glutaraldehyde until further processing. Samples were rinsed thrice with PIPES [piperazine-*N*,*N*′-*bis*(ethanesulfonic acid)] buffer (catalog number P6757; Sigma-Aldrich), followed by a second fixation in 1% osmium tetroxide (OsO4) (catalog number 75632; Sigma-Aldrich) for 1 h at RT, first embedding into agar to reduce sample loss. Pellets were then dehydrated using ethanol (EtOH) (50, 70, and 90% for 10 min each and 3 times at 100% for 20 min). After dehydration, samples were infiltrated with a 1:1 epoxy resin-propylene oxide mixture overnight at 37°C. The next day, pellets were infiltrated with fresh epoxy resin for 1 h at 37°C, followed by polymerization at 60°C overnight in plastic molds. Samples were viewed using a Philips CM100 transmission electron microscope equipped with a TENGRA 2.3-megapixel by 2.3-megapixel camera.

### Anti-TB agent challenge assays.

To evaluate the mechanisms of action of anti-TB agents on bacterial growth, a 0.75 MIC challenge assay was conducted to observe the growth response of M. smegmatis mc^2^ 155. A 1% inoculum in the presence of 0.75 MIC of each anti-TB agent, alone or in combination, was cultured in 10 ml Middlebrook broth medium at 37°C. After 5 days, the bacteria were processed and subjected to *trans*-1,6-diphenyl-1,3,5-hexatriene (DPH) fluorescence assay and laurdan fluorescence emission and nuclear magnetic resonance (NMR) metabolomics analyses.

### DPH fluorescence assay.

An M. smegmatis mc^2^ 155 bacterial suspension was harvested and fixed using 0.25% formaldehyde for 1 h at room temperature. After fixing, the bacterial cells were centrifuged at 5,000 rpm and 4°C for 8 min and then washed with 5 ml PBS once. Bacteria were resuspended in PBS and stained with 2.5 µM DPH fluorescence probe. All tubes were wrapped with foil to prevent light degradation of the dye and incubated at 37°C for 30 min. Single-wavelength measurements were taken at 430-nm emission and 358-nm excitation using a Varian Cary Eclipse fluorescence spectrophotometer (Agilent, Santa Clara, CA). The spectral bandwidth of the emission monochromator and the measurement of emission spectra were set at 10 nm. Steady-state fluorescence anisotropy, *r*, was calculated with the formula *r* = (*I*_VV_ − *GI*_VH_)/[*I*_VV_ − 2(*GI*_VH_)], where *I*_VV_ and *I*_VH_ are the parallel and perpendicular polarized fluorescence intensities measured with the vertically polarized excitation light, *I*_HV_ and *I*_HH_ are the same fluorescence intensities measured with the excitation light horizontally polarized, and *G* is the monochromator grating correction factor given by *G* = *I*_HV_/*I*_HH_.

### Laurdan fluorescence assay.

M. smegmatis mc^2^ 155 bacterial pellets were harvested, fixed, and washed as described above and then stained with 2.5 µM laurdan (6-dodecanolyl-*N*,*N*-dimethyl-2-napthtylamine) fluorescence probe. All tubes were wrapped with foil to prevent light degradation of the dye and incubated at 37°C for 1 h. Twenty emission wavelength spectra, from 400 to 600 nm, were obtained with excitation at 350 nm on the same fluorescence spectrophotometer. The general polarization (GP) value corresponding to the fluidity of the membrane was calculated using the formula GP = (*I*_440_ − *I*_490_)/(*I*_440_ + *I*_490_), where *I*_440_ and *I*_490_ are the emission intensities at the corresponding wavelengths.

### NMR metabolomics.

An M. smegmatis mc^2^ 155 bacterial suspension was pelleted by centrifugation at 5,000 rpm, 4°C, for 8 min. Supernatant was collected and filtered through 0.22-µm membrane filters to remove any remaining bacterial cells. The bacterial pellet was washed twice and resuspended in PBS. All supernatant samples were frozen at −80°C, and pellets were snap-frozen in liquid nitrogen before freeze drying using an Alpha 1-2 LDplus freeze dryer (Martin Christ, Germany). After lyophilization, the supernatant was rehydrated by using 10% D_2_O containing 3-(trimethylsilyl)propionic-2,2,3,3-*d*_4_ acid sodium salt (TSP-*d*_4_) to provide a deuterium lock signal with a reference signal. Samples were then transferred to NMR tubes. ^1^H NMR spectra were recorded on a Bruker Avance II 700 NMR spectrometer (Bruker BioSpin, Coventry, United Kingdom) equipped with a 5-mm helium-cooled quadruple resonance cryoprobe, with nine sample replicates tested per condition and all kept at 4°C. One-dimensional (1-D) spectra were recorded under automation at 298 K using a Carr-Purcell-Meiboom-Gill presaturation (cpmgpr1) pulse sequence. Spectra were acquired with 64 transients, a spectrum width of 20.1 ppm, and 65,536 data points. For the freeze-dried bacterial pellets, 40 µl of D_2_O was used for rehydration. The resuspended samples were placed in Kel-F inserts and then in the 4-mm zirconia magic angle spinning (MAS) rotor and preserved with a Kel-F cap (Bruker, Rheinstetten, Germany). Data acquisition was performed on a 600 MHz Bruker Avance III spectrometer equipped with a 4-mm HR-MAS probe, keeping the temperature at 310 K. The spinning speed was 5 kHz. ^1^H NMR spectra were collected with a pulse-acquire with presaturation (zgpr) pulse sequence, a spectrum width of 12.01 ppm, and a relaxation delay (d1) of 3 s. The total acquisition time was 1.14 s, with a prescan delay of 6.5 µs. A total of 128 scans were used to obtain each of the NMR spectra. The free induction decay was multiplied by an exponential function with line broadening of 0.3 Hz. To aid the assignment of metabolite resonance, correlation spectroscopy (COSY) (cosygpprqf) spectra were acquired for a subset of samples. All peak positions were measured relative to the methyl peak of TSP-*d*_4_, set to 0.0 ppm. Phase correction of spectra was performed manually, and automatic baseline correction was applied.

### Data analysis and statistics.

All microbial data presented in this study were statistically analyzed by using GraphPad Prism (version 5.01 for Windows; GraphPad Software, Inc., La Jolla CA). One-way analysis of variance (ANOVA) was performed, followed by Dunnett’s multiple-comparison test with a value of *P* ≤ 0.05 to establish statistical significance. Sigmoidal dose-response (variable slope) was used for fitting of growth inhibition calculations. All experiments were performed at least 3 times.

For metabolomics data, peak assignment was performed by comparing chemical shift values and multiplicities from J-resolved NMR spectra to values from the Biological Magnetic Resonance Bank (BMRB) (48), COSY spectra, and NMR metabolite templates in Chenomix NMR suite software (Chenomx, Inc., Edmonton, Canada). Multivariate data analysis was based on previously published work (49). Principal-component analysis (PCA) and orthogonal projections to latent structures discriminant analysis (OPLS-DA) were done using MVAPACK (50) and software developed in our previous study (49). Spectra were subjected to probabilistic quotient normalization (PQN) and autoscaled (51). The icoshift algorithm was applied for further alignment, and an optimized bucketing algorithm with a 0.005-ppm bin size was employed for bucketing. Cross-validation was performed as described previously (49), with the modifications that 85% of the samples were used as a training set and the remaining 15% as a test set and a maximum of 5 components were used. Double cross-validation was repeated 1,000 times, producing 7 × 1,000 models. The reference *Q*^2^ value was generated after the known or random class assignment process, providing a measure of goodness of fit after cross-validation, generally considered to be good above 0.5 (52, 53). The *Q*^2^ value quoted herein (Table 3) is the mean value of all models and was compared between the genuine and permutated class assignments in each case. Heatmaps with Euclidian distance-hierarchical cluster (HCL) analysis were generated by using MultiExperiment Viewer (MeV) software.

### Volcano plots and univariate analysis of metabolomics data.

For volcano plots, significant results from PLS or OPLS-DA analysis justified univariate analysis of peaks associated with predictive value in the multivariate models. Therefore, the integrals of assigned NMR peaks were analyzed using nonparametric univariate methods. Volcano plots were used to compare the fold changes in metabolite values between two conditions. Fold change was calculated as the ratio between the results for the treatment condition and the control (treatment/control). The Mann-Whitney *U* test was used to compare mean values, and the associated *P* values were false discovery rate adjusted using the Benjamini-Hochberg method (α = 0.05). Volcano plots were generated using custom scripts in Python with the Numpy, Pandas, Matplotlib, and Seaborn packages.

### PLS regression.

PLS regression was performed in Python using the PLSRegression function in the scikit-learn software package. The initial model assessment determined that only one component was necessary for best model performance. An increased number of components led to a decrease in *Q*^2^. Monte Carlo cross-validation of models was performed by randomly splitting data into 70/30 training/test set splits. Model generation and assessment were repeated 1,000 times to avoid bias by sample separation, and *R*^2^ and *Q*^2^ values were calculated to assess model performance. The *R*^2^ metric demonstrates how well the model describes the training data set, while *Q*^2^ is a metric of how well the model predicts the test set.
