# Supplementary figures and images for: Rifampin- or Capreomycin-Induced Remodeling of the Mycobacterium smegmatis Mycolic Acid Layer Is Mitigated in Synergistic Combinations with Cationic Antimicrobial Peptides
Source: mSphere. 2018 Jul 18;3(4):e00218-18. doi: 10.1128/mSphere.00218-18 (PMC6052339; doi:10.1128/mSphere.00218-18)

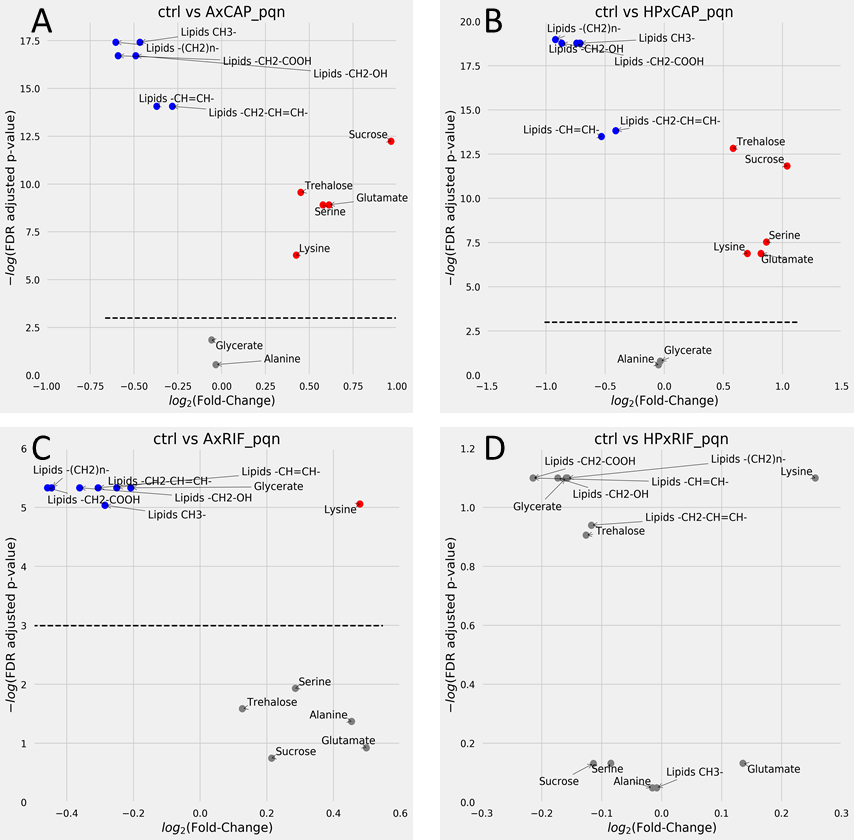

Supplement: FIG S1 [file sph004182592sf1.tif]

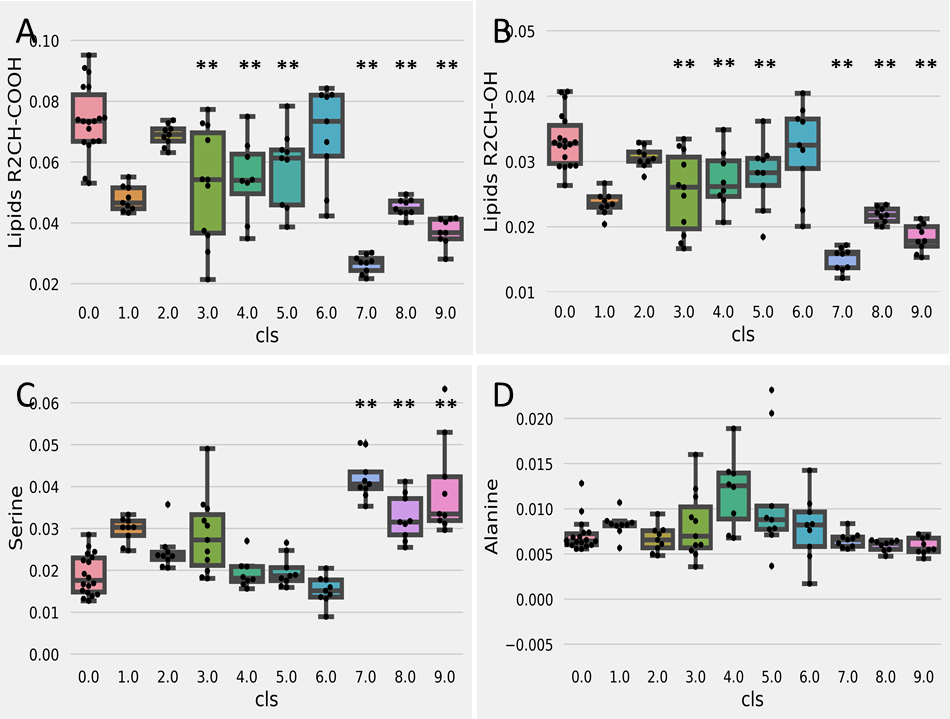

Supplement: FIG S2 [file sph004182592sf2.tif]

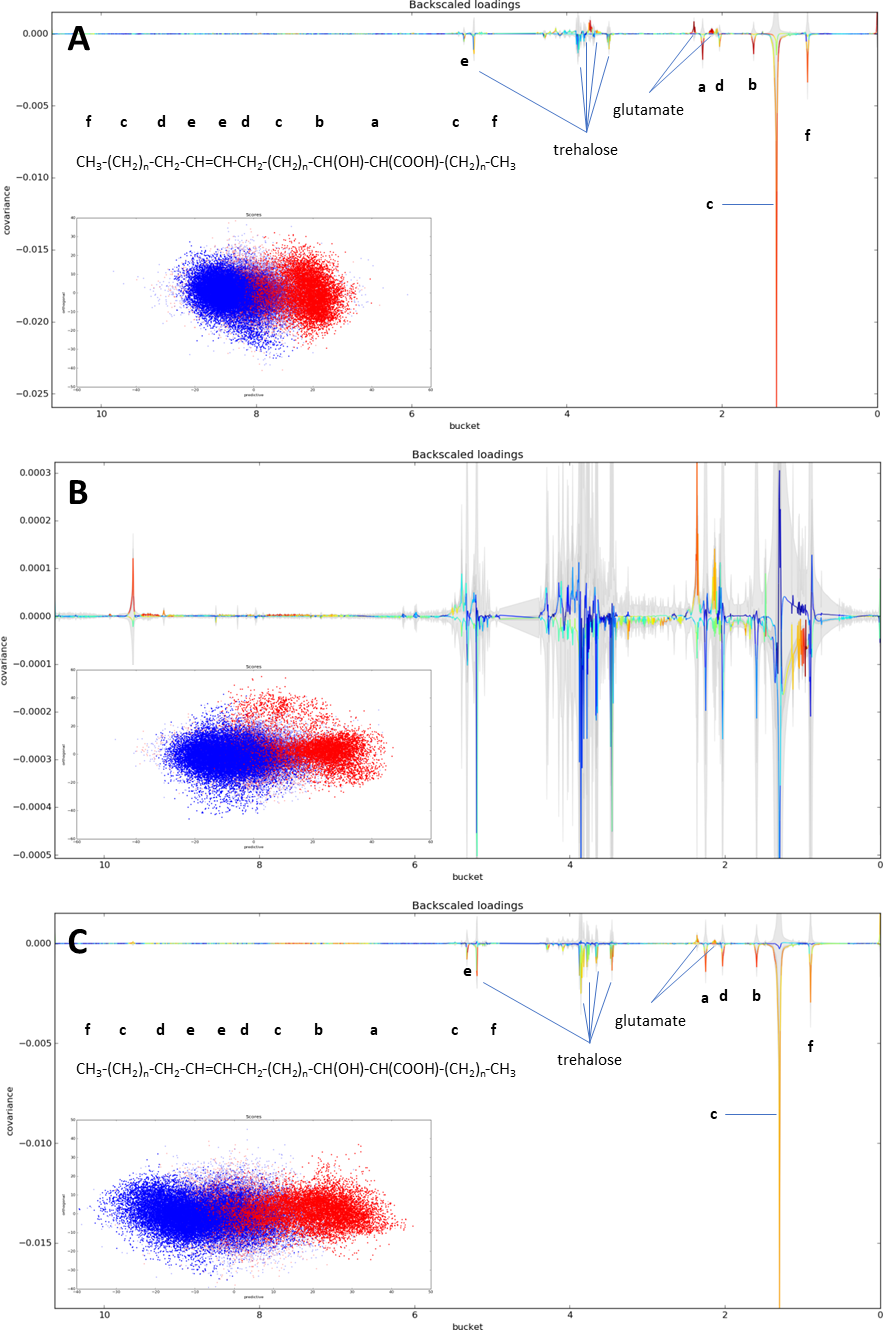

Supplement: FIG S3 [file sph004182592sf3.tif]

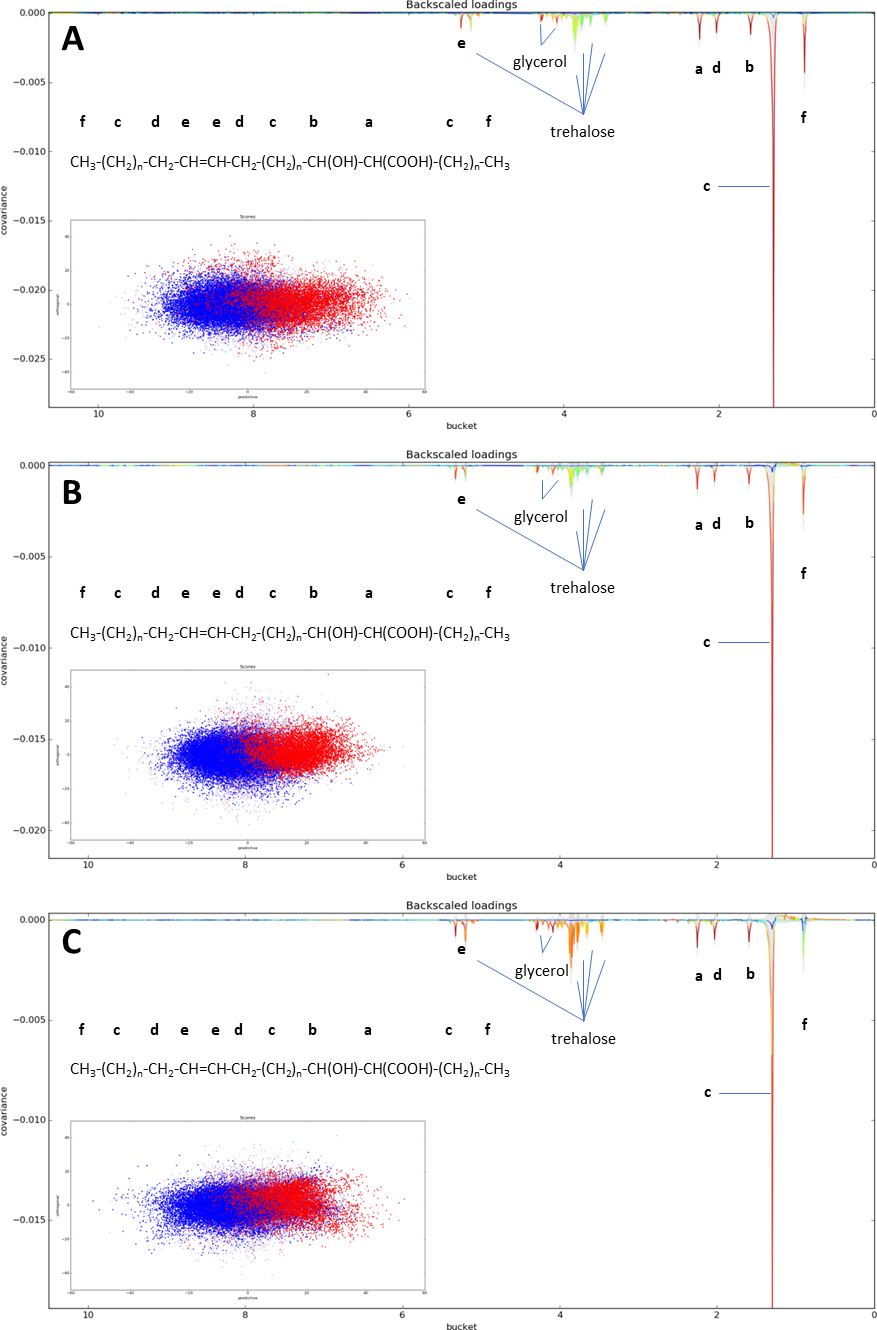

Supplement: FIG S4 [file sph004182592sf4.tif]

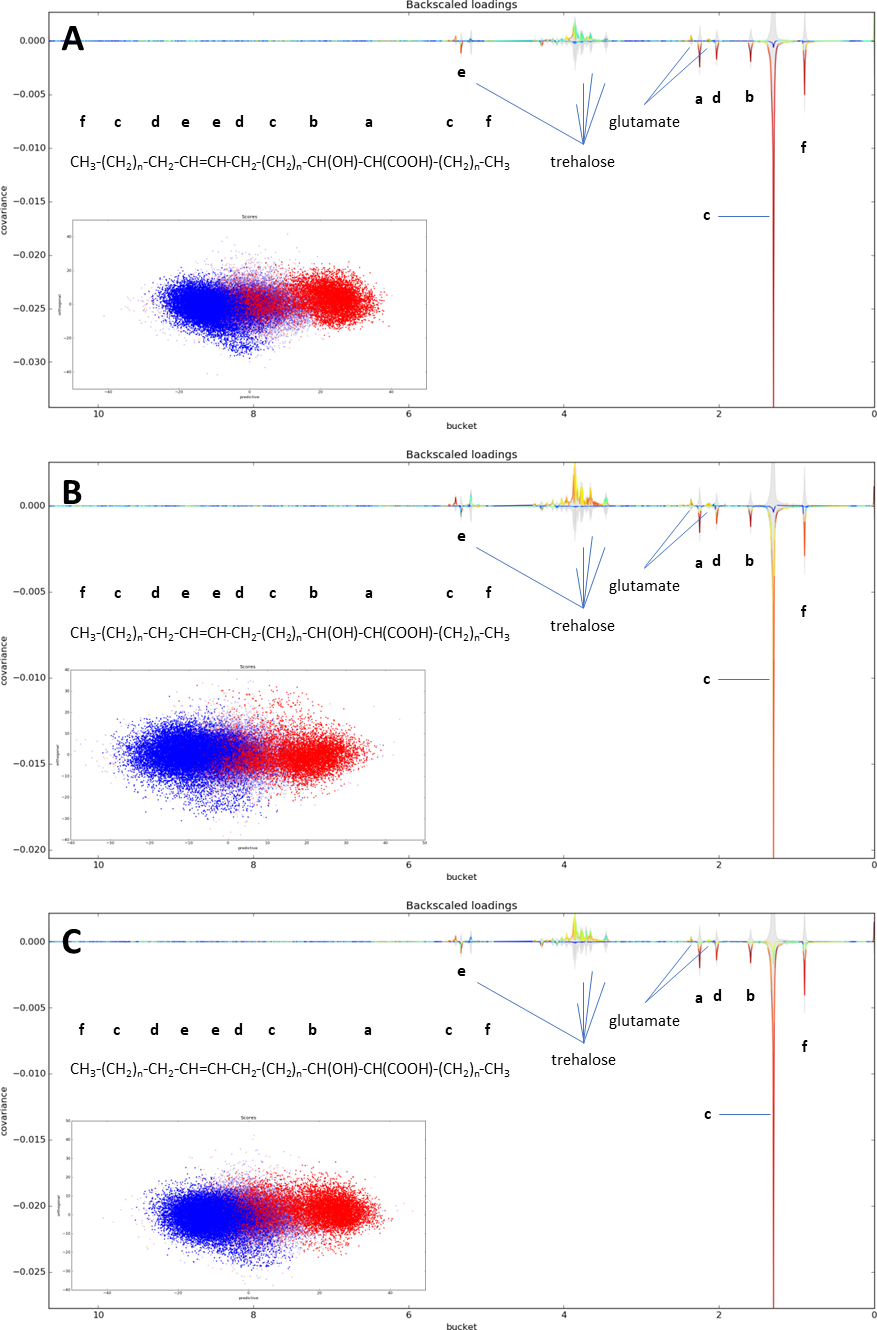

Supplement: FIG S5 [file sph004182592sf5.tif]

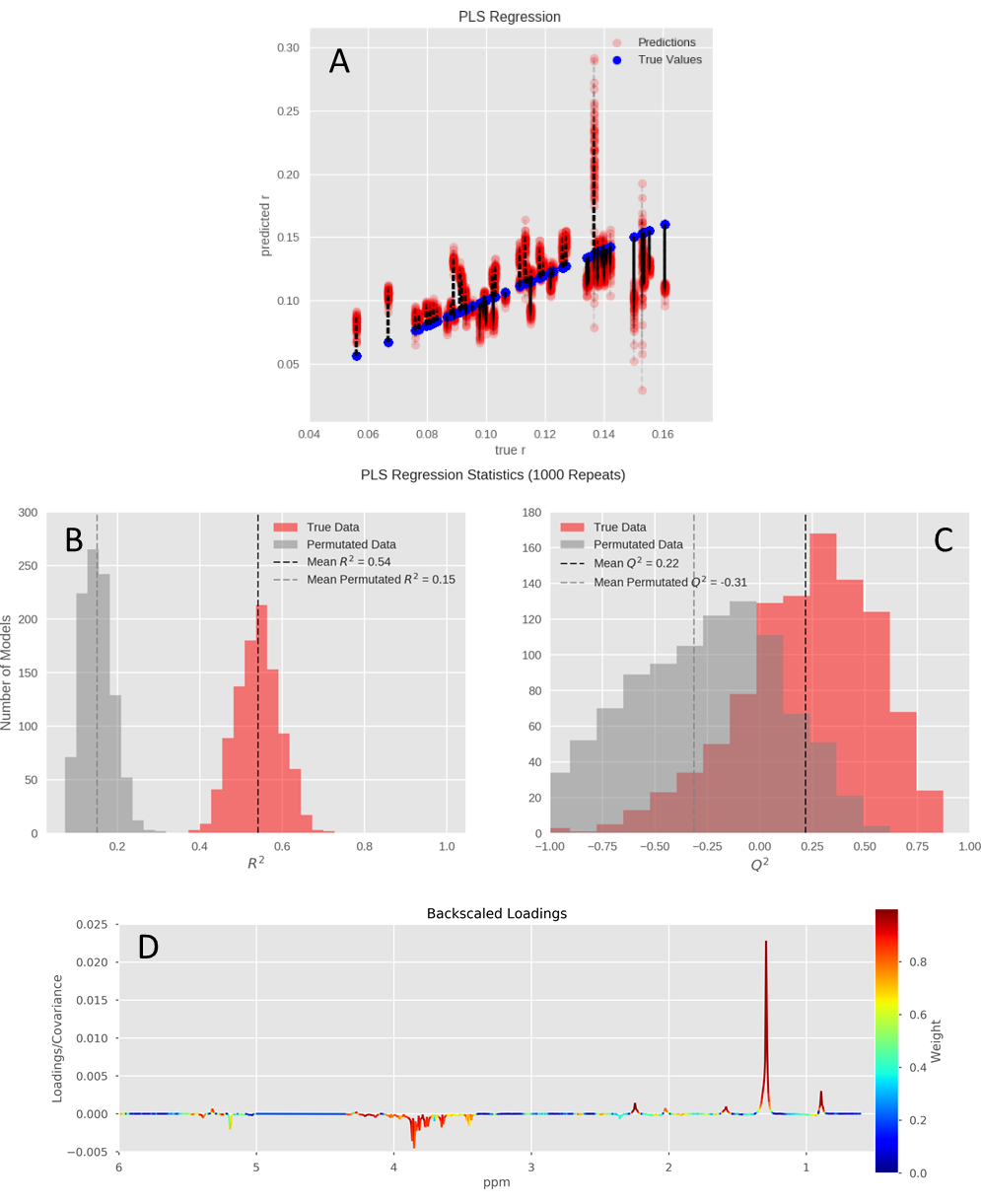

Supplement: FIG S6 [file sph004182592sf6.tif]

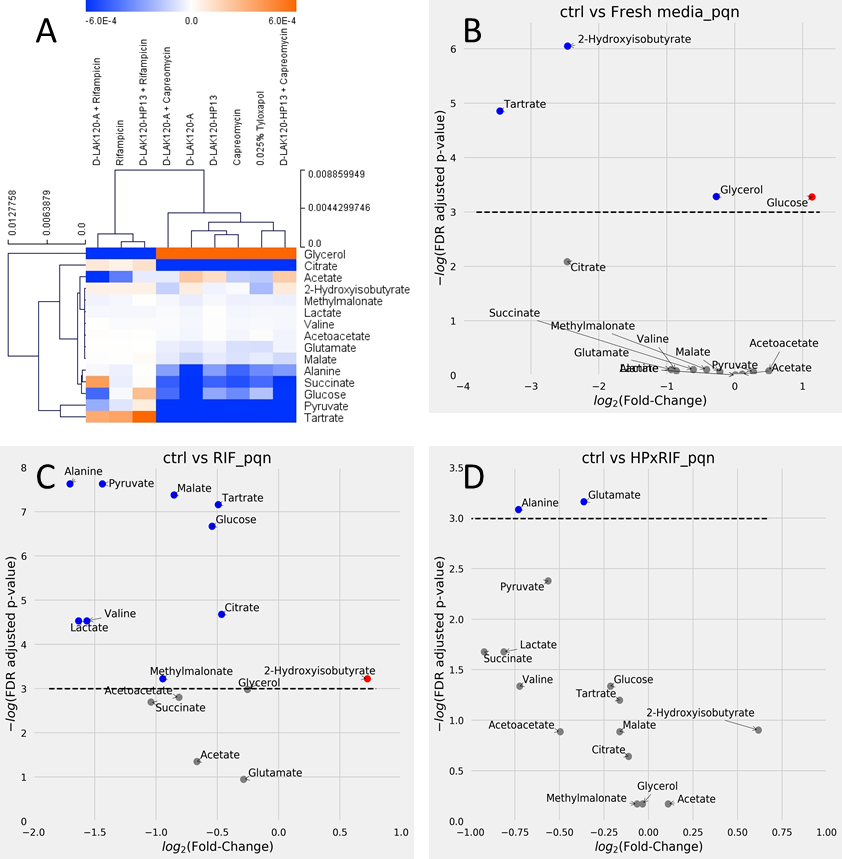

Supplement: FIG S7 [file sph004182592sf7.tif]
